# Visualizing complex feature interactions and feature sharing in genomic deep neural networks

**DOI:** 10.1186/s12859-019-2957-4

**Published:** 2019-07-19

**Authors:** Ge Liu, Haoyang Zeng, David K. Gifford

**Affiliations:** Computer Science and Artificial Intelligence Laboratory, Massachusetts Institute of Technology, Cambridge, Massachusetts, USA

**Keywords:** Visualization, Deep neural networks, Combinatorial interactions

## Abstract

**Background:**

Visualization tools for deep learning models typically focus on discovering key input features without considering how such low level features are combined in intermediate layers to make decisions. Moreover, many of these methods examine a network’s response to specific input examples that may be insufficient to reveal the complexity of model decision making.

**Results:**

We present DeepResolve, an analysis framework for deep convolutional models of genome function that visualizes how input features contribute individually and combinatorially to network decisions. Unlike other methods, DeepResolve does not depend upon the analysis of a predefined set of inputs. Rather, it uses gradient ascent to stochastically explore intermediate feature maps to 1) discover important features, 2) visualize their contribution and interaction patterns, and 3) analyze feature sharing across tasks that suggests shared biological mechanism. We demonstrate the visualization of decision making using our proposed method on deep neural networks trained on both experimental and synthetic data. DeepResolve is competitive with existing visualization tools in discovering key sequence features, and identifies certain negative features and non-additive feature interactions that are not easily observed with existing tools. It also recovers similarities between poorly correlated classes which are not observed by traditional methods. DeepResolve reveals that DeepSEA’s learned decision structure is shared across genome annotations including histone marks, DNase hypersensitivity, and transcription factor binding. We identify groups of TFs that suggest known shared biological mechanism, and recover correlation between DNA hypersensitivities and TF/Chromatin marks.

**Conclusions:**

DeepResolve is capable of visualizing complex feature contribution patterns and feature interactions that contribute to decision making in genomic deep convolutional networks. It also recovers feature sharing and class similarities which suggest interesting biological mechanisms. DeepResolve is compatible with existing visualization tools and provides complementary insights.

**Electronic supplementary material:**

The online version of this article (10.1186/s12859-019-2957-4) contains supplementary material, which is available to authorized users.

## Background

Deep learning has proven to be powerful on a wide range of tasks in computer vision and natural language processing [1–5]. Recently, several applications of deep learning in genomic data have shown state of art performance across a variety of prediction tasks, such as transcription factor (TF) binding prediction [6–9], DNA methylation prediction [10, 11], chromatin accessibility [12], cell type-specific epigenetic[13], and enhancer-promoter interaction prediction [14] However, the composition of non-linear elements in deep neural networks makes interpreting these models difficult [15], and thus limits model derived biological insight.

There have been several attempts to interpret deep networks trained on genomic sequence data. One approach scores every possible single point mutation of the input sequence [6]. Similarly, DeepSEA analyzed the effects of base substitutions on chromatin feature predictions [8]. These ‘in silico saturated mutagenesis’ approaches reveal individual base contributions, but fail to identify higher order base interactions as they experience a combinatorial explosion of possibilities as the number of mutations increases.

The second class of efforts to visualize neural networks uses internal model metrics such as gradients or activation levels to reveal key input features that drive network decisions. Zeiler et al. used a de-convolutional structure to visualize features that activate certain convolutional neurons [16, 17]. Simonyan et al. proposed saliency maps which use the input space gradient to visualize the importance of pixels to annotate a given input [18]. Simonyan’s gradient based method inspired variants, such as guided back-propagation [19] which only considers gradients that have positive error signal, or simply multiplying the gradient with the input signal. Bach et al. [20] proposed layer-wise relevance propagation to visualize the relevance of the pixels to the output of the network. Shrikumar et al. [21] proposed DeepLIFT which scores the importance of each pixel, by defining a ‘gradient’ that compares the activations to a reference sequence, which can resolve the saturation problem in certain types of non-linear neuron paths. LIME [22] creates a linear approximation that mimics a model on a small local neighborhood of a given input. Other input-dependent visualization methods include using Shapley values [23], integrated gradients [24], or maximum entropy [25]. While these methods can be fine-grained, they have the limitation of being only locally faithful to the model because they are based upon the selection of an input. The non-linearity and complex combinatorial logic in a neural network may limit network interpretation from a single input. In order to extract generalized class knowledge, unbiased selection of input samples and non-trivial post-processing steps are needed to get a better overall understanding of a class. Moreover these methods have the tendency to highlight existing patterns in the input due to the nature of their design, while the network could also make decisions based on patterns that are absent.

Another class of methods for interpreting networks directly synthesize novel inputs that maximize the network activation, without using reference inputs. For example, Simonyan et al. [18] uses gradient ascent on input space to maximize the predicted score of a class, and DeepMotif [26] is an implementation of this method on genomic data. These gradient ascent methods explore the input space with less bias. However their main focus is generating specific input patterns that represent a class without interpreting the reasoning process behind these patterns. Moreover when applied to computer vision networks the images they generate are usually unnatural [27]. Thus gradient methods are typically less informative than input-dependent methods for visual analysis. The unnaturalness of gradient images can be caused by the breaking of spatial constraints between convolutional filters.

While all of the above methods aim to generate visual representations in input space, few have focused on the interpretation of *feature maps* that encode how input features are combined in subsequent layers. In genomic studies, lower level convolutional filters capture short motifs, while upper layers learn the combinatorial ‘grammar’ of these motifs. Recovering these combinatorial interactions may reveal biological mechanism and allow us to extract more biological insights.

Here we introduce DeepResolve, a gradient ascent based visualization framework for feature map interpretation. DeepResolve computes and visualizes *feature importance maps* and *feature importance vectors* which describe the activation patterns of channels at a intermediate layer that maximizes a specific class output. We show that even though gradient ascent methods are less informative when used to generate representations in input space, gradient methods are very useful when conducted in feature map space as a tool to interpret the internal logic of a neural network. By using multiple random initializations and allowing negative values, we explore the feature space efficiently to cover the diverse set of patterns that a model learns about a class. A key insight of DeepResolve is that the visualization of the diverse states of an internal network layer reveals complex feature contribution patterns (e.g. negatively contributing or non-linearly contributing features) and combinatorial feature interactions which can not be easily achieved using other existing visualization tools that operate on input space. The correlation of the positive feature importance vector for distinct classes reveals shared features between classes and can lead to an understanding of shared mechanism. Our automatic pipeline is capable of generating analysis results on feature importance, feature interactions and class similarity, which can be used for biological studies. DeepResolve requires no input dataset or massive post-processing steps and thus is spatially efficient.

## Methods

### Visualizing feature importance and combinatorial interactions

#### Class Specific Feature Importance Map and Feature Importance Vector

Unlike methods which use gradient-ascent to generate sequence representations in the input layer[18, 26], DeepResolve uses gradient-ascent to compute a class-specific optimal feature map *H*_*c*_ in a chosen intermediate layer *L*. We maximize the objective function:

$$H_{c} = \mathop{\arg\max}\limits_{H} S_{c}(H)-\lambda||H||_{2}^{2}$$

*S*_*c*_ is the score of class *c*, which is the *c*-th output in the last layer before transformation to probability distribution (before sigmoid or soft-max). The class-specific optimal feature map is $H_{c} \in \mathcal {R}^{K \times W}$ for a layer having *K* feature maps of size *W* (*W* is the width of the feature maps after max-pooling and *W*=1 when global max-pooling is used). *K* is the number of sets of neurons that share parameters. Each set of neurons that share parameters is called a channel, and each channel captures unique local features within a receptive field. We name *H*_*c*_ a *feature importance map* (FIM) for class *c*, and each map entry $(H^{k}_{i})_{c}$ evaluates the contribution of a neuron from channel *k* in a specific position *i* in a layer. When local max-pooling is used, a FIM is capable of capturing the spatial pattern of feature importance within each channel. In typical biological genomic neural networks, spatial specificity is in general low because of the stochasticity in input feature locations. Therefore we compute a feature importance score $\phi ^{k}_{c}$ for each of the *K* channels by taking the spatial average of the feature importance map (*H*^*k*^)_*c*_ of that channel. These scores collectively forms a *feature importance vector* (FIV) $\Phi _{c}=((\phi ^{1}_{c}),(\phi ^{2}_{c}),\ldots,(\phi ^{k}_{c}))$:

$$\phi^{k}_{c}=\frac{1}{W}\sum\limits_{i=1}^{W} (H^{k}_{i})_{c}$$

Note that although the natural domain of feature map is $\mathbb {R}^{+}_{0}$ if ReLU units are used, we allow FIMs to have negative values during gradient ascent so as to distinguish channels with negative scores from those with close to zero scores. The feature importance score for each channel represents its contribution pattern to the output prediction and a channel can contribute positively, negatively or trivially. Positive channels usually associate with features that are ’favored’ by the class, whereas negative channels represents features that can be used to negate the prediction. We found that negative channels contain rich information about the reasoning of network decisions. Negative channels can capture patterns that do not exist in positive samples or non-linearly interacting patterns.

#### Visualizing complex feature contribution patterns and interactions

Since deep neural networks have the capacity to learn multiple patterns for a single class, the learned function space can be multimodal. Moreover, the channels may contribute differently in different modes and their contributions may condition on the other channels, which indicate complex feature contribution patterns and interactions. However an input dependent visualization method usually explores only one of the modes when a specific sample is given. To explore the optimums in the space more efficiently, we repeat gradient ascent multiple times (*T* times) for each target class *c* using different random initialization sampled from normal distribution. This generates an ensemble of FIMs $\{H^{t}_{c}\}$ and FIVs $\{\Phi _{c}^{t}\}$ for each class.

To reduce the effect of bad initializations we weight each gradient ascent result using the output class score. We add an offset to the scores such that all trials have non-negative weights. The ensemble of FIVs exhibits diverse representations of feature space patterns learned by the corresponding class, with some channels having more inconsistent contribution than others. We evaluate the weighted variance of the feature importance score of each channel *k* in the ensemble, and use it as a metric to evaluate the inconsistency level (IL) of the channel *k* for target class *c*:

$$IL_{c}^{k}=\text{Var}[(\phi_{c}^{k})^{t}]$$

**Fig. 1:**
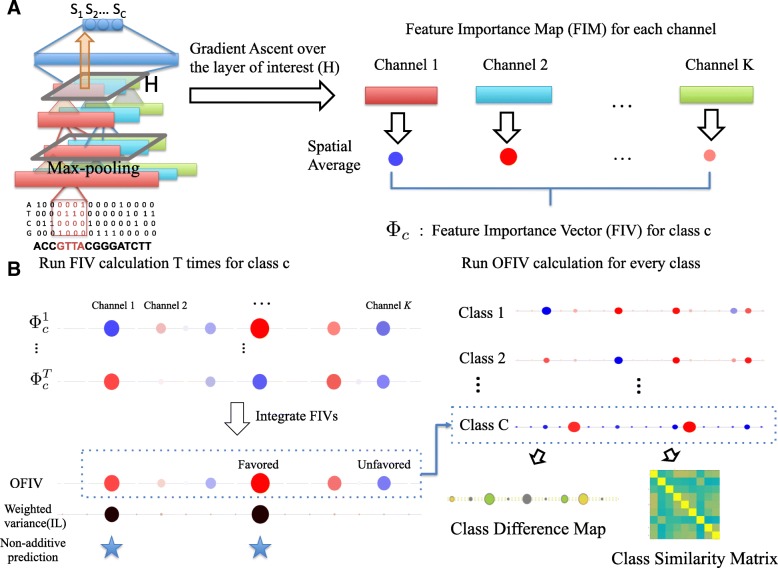
Illustration of DeepResolve’s working flow. **a** Feature Importance Vectors calculation. After a network is trained and a intermediate layer is selected, DeepResolve first computes the feature importance maps (FIM) of each of the channels using gradient ascent. Then for each channel, the Feature Importance Vector (FIV) score is calculated as the spatial average of its FIM scores. **b** Overall Feature Importance Vector calculation. For each class, DeepResolve repeats the FIV calculation *T* times with different random initializations. The weighted variance over the *T* times is then calculated as an indicator of inconsistency level (IL) of each channel. A Gaussian Mixture Model is trained on IL scores to determine the non-additiveness of a channel. For each channel, the *T* FIVs are combined with the reference to the inconsistency level to generate an Overall Feature Importance Vector (OFIV) which summarizes all ‘favored’ and ‘unfavored’ patterns of a class. Finally, we use the non-negative OFIVs of each class to analyze class similarity and the OFIVs to analyze class differences

**Fig. 2:**
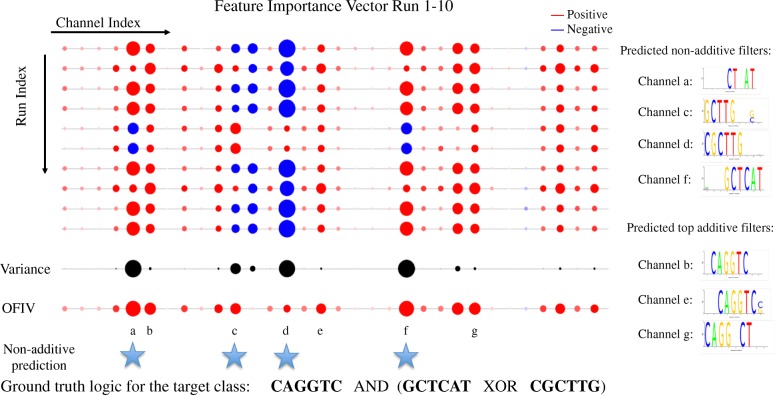
Illustration of the generation of OFIV from FIVs generated by all 10 runs of gradient ascent in synthetic data set I. Red circles on the X-axis represent positive channels and blue circles represent negative channels. Circle size is proportional to the absolute FIV value. The weighted variance (IL score) of each channel is plotted below the FIVs, where the darkness and circle size is proportional to the variance. The OFIV is visualized below, where the circle size reflect the overall importance score of a channel. The channels that are predicted as non-additive by the Gaussian Mixture Model fitted on the IL scores are labeled by a star. A seqlogo visualization of the filter weight is plotted next to the corresponding channel. Filter {a,f} and {c,d} which capture sequences that involve in XOR logic are correctly predicted as non-additive. Among the remaining filters, the top-OFIV ones {b,c,g} which capture the sequence that involve in AND logic are correctly predicted as additive

Channels with a low inconsistency level contribute to the output either positively, negatively, or not at all. We define this type of channel as a *additive channel* because their contributions can be combined additively (e.g. AND/OR/NOT logic). We define channels with high inconsistency as *non-additive channels* since their contribution is inconsistent and usually conditioned on the other channels (e.g. XOR logic). We visualize the signs and magnitudes of FIV scores of the entire ensemble of FIVs as shown in Figs. 1 and 2. In this way both individual and combinatorial interactions between channels can be easily perceived. In the results section below we show the effectiveness of this visualization using synthesized data in discovering XOR logic where two channels always have opposite contributions.

#### Summarizing feature contributions using Overall Feature Importance Vector

We summarize the contribution of a feature using an *overall feature importance vector* (OFIV) $\bar {\Phi }_{c}$ that takes into account the rich information of the magnitude and direction of the feature contribution embedded in the ensemble of FIVs.

We first calculate the weighted variance of the FIVs for each channel to get the inconsistency level (IL). Three Gaussian mixture models with the number of components varying from one to three are fitted over the IL scores to account for channels that are additive and non-additive. The final number of mixture components is picked to minimize the Bayesian Information Criterion (BIC).

We next categorize the channels by IL score and the sign of contribution to calculate category-specific OFIVs that properly characterizes the feature importance. The channels in the mixture component with the lowest mean are considered as either additive or unimportant. The remaining mixture components (if any) are considered as non-additive channels and can be further categorized by whether the sign of its FIVs in the ensemble is consistent. For channels considered as additive, unimportant, or non-additive with consistent sign, the OFIV is calculated as the weighted average of its scores across all FIVs. For channels considered as non-additive with inconsistent sign, the OFIV is calculated as the weighted average of the positive FIVs in the ensemble to reflect the feature contribution in cases where the channel is not used to negate the prediction.

Visualizing OFIVs and IL scores together, we recover both the importance level of different features and the presence of non-additive channels. We automatically produce a list of important features, and a list of non-additive features that are highly likely to involved in complex interactions.

### Visualizing feature sharing and class relationship

The weight sharing mechanism of multi-task neural networks allows the reuse of features among classes that share similar patterns. In past studies, the weight matrix in the last layer has been used to examine class similarity. However, this is potentially problematic because the high-level features in a network’s last layer tend to be class-specific. This method also fails to discover lower level feature sharing between classes that are rarely labeled positive together. Using OFIVs proposed above, we revisit the feature sharing problem to enable the discovery of lower-level feature sharing when the class labels are poorly correlated.

We observe that the network learns to use negative channels to capture class-specific patterns in other classes as a process of elimination to maximize the prediction accuracy. This potentially increases the distance of those classes in hidden space despite the fact that they may share other features. Thus, while neurons with both strong positive and negative OFIV scores are potentially important for making the prediction, only the ones with positive OFIV scores are truly associated with the target class. Inspired by this finding, we introduce a class similarity matrix *A* by taking pair-wise Pearson correlation of non-negative OFIV of all the classes.

$$A_{C_{i}C_{j}}=\frac{\text{Cov}\left(\bar{\Phi}_{c_{i}}^{+},\bar{\Phi}_{c_{j}}^{+}\right)}{\sigma_{\bar{\Phi}_{c_{i}}^{+}} \sigma_{\bar{\Phi}_{c_{j}}^{+}}}$$

$\bar {\Phi }_{c}^{+}$ encodes the composition of all positive contributing features for a given class in intermediate layer. By taking the difference of OFIV of a pair of classes, we can also generate a class difference map.

$$D_{C_{i}C_{j}}=\bar{\Phi}_{c_{i}}-\bar{\Phi}_{c_{j}}$$

This map highlights features that are favored by one class but not favored by the other. This is especially helpful when studying cell-type specific problems where a key feature deciding differential expression or binding in different cell type might be crucial.

### Implementation details

We trained all of our models with Keras version 1.2 and the DeepSEA network is downloaded from the official website. We convert the torch DeepSEA model into Caffe using torch2caffe and the resulting model has same performance as the original network. We implemented DeepResolve for both Caffe and Keras. As baselines, we implemented saliency map and DeepMotif in Keras, and used DeepLIFT v0.5.1 for generating DeepLIFT scores.

## Results

### Synthetic datasets

#### Recovering important features and combinatorial interactions

We tested if FIVs would highlight important features and identify complex feature interactions in a synthetic data set which contains both additive and non-additive combinatorial logic. Synthetic dataset I contains 100,000 DNA sequences, each containing patterns chosen from CGCTTG, CAGGTC and GCTCAT in random positions. We label a sequence 1 only when CAGGTC and one of (GCTCAT, CGCTTG) present, and otherwise 0. This is the combination of AND logic and XOR logic. We also include 20,000 sequences that are totally random and label them as 0. We trained a convolutional neural network with a single convolutional layer with 32 8bp filters and local max-pooling with stride 4, followed by a fully connected layer with 64 hidden units. 20% of the data were held out as a test set and the resulting test AUC was 0.985. We applied DeepResolve on the layer in between convolutional layer and fully connected layer, and each channel correspond to a convolutional filter that can be visualized as Position Weight Matrix after normalization.

As shown in Fig. 2, when ranked by OFIV, the top filters predicted to be non-additive capture CGCTTG and GCTCAT, the pair of motifs that non-linearly (XOR) interact with each other. The top filters predicted to be additive characterize CAGGTC, the motif that additively (AND) interacts with the other ones. Furthermore, the FIVs correctly unveil the non-additive XOR interaction between GCTCAT and CGCTTG as the corresponding filters tend to have opposite signs all the time. The optimal number of Gaussian mixture components of the IL score is 3 (Additional file 1: Figure S1), indicating the existence of non-additiveness.

**Fig. 3:**
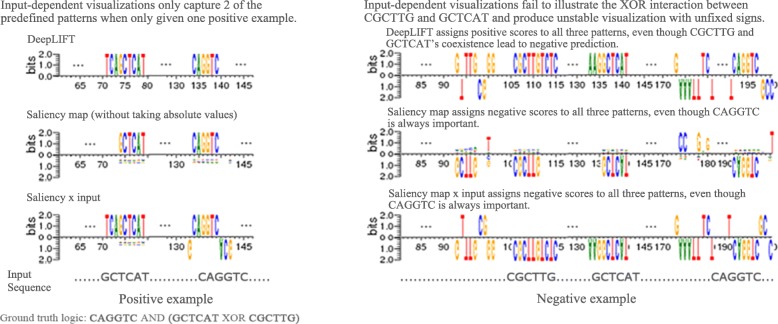
Input-dependent visualizations produce unstable results on XOR logic and fail to capture the XOR interaction. Three types of input-dependent visualizations on example positive and negative sequence from synthetic data set I. The visualization using positive example (left) only highlight two of the 3 predefined patterns because a positive sample can only contain one of GCTCAT,CGCTTG, while the third pattern will always be missing. When using negative example which contains all three patterns as the input, all of the methods assign either all positive or all negative scores to the three patterns (right), failing to capture the XOR interaction between GCTCAT and CGCTTG. The saliency methods predict negative score for CAGGTC, a pattern that should always exists in positive examples, suggesting that these methods are not stable enough when dealing with complex logic

We further compared three types of input-dependent visualizations: DeepLIFT, saliency map, and saliency map multiplied by input. For our comparison we used positive and negative examples from synthetic dataset I, where the positive example contains GCTCAT and CAGGTC, and the negative example contains all three patterns. The network prediction on these examples are correct, suggesting that it has learned the XOR logic. Note that the original saliency map takes the absolute value of the gradients which never assign negative scores and thus limits the interpretation of the internal logic of a network. Thus we used the saliency map without taking the absolute value to allow for more complex visualizations. We compute attribution scores for each base pair in the input with regard to the positive class’s softmax logit. As shown in Fig. 3, the visualization on positive example can be biased by the choice of input since only the 2 patterns that present in the input will be highlighted and the third pattern is always missing. On the other hand, when a negative example is used as input, all three methods assign scores with the same signs to all three patterns, making the XOR logic indistinguishable from AND logic. DeepLIFT assigns positive score to both GCTCAT and CAGGTC even though their co-existence lead to negative prediction. Moreoever, the saliency methods incorrectly assign negative score to CAGGTC which is designed to always exists in positive class. This shows that saliency methods can be unstable in attributing positively contributing patterns when complex non-linear logic exists.

#### Recovering class relationships

We synthesized dataset II to test our ability to discover feature sharing when the labels are poorly correlated. Synthetic dataset II has 4 classes of DNA sequences with one class label assigned to each sequence. Class 1 contains GATA and CAGATG, class 2 contains TCAT and CAGATG, Class3 contains GATA and TCAT, while class 4 only contains CGCTTG. The introduced sequence patterns are deliberately selected such that three of the classes share half of their patterns, while class 4 is totally different. These four classes are never labeled as 1 at the same time, thus the labels yield zero information about their structural similarities. We trained a multi-task CNN with a single convolutional layer that has 32 8bp long filters, one fully connected layer with 64 hidden neurons, and a four-neuron output layer with sigmoid activation to predict the class probability distribution. The test AUC is 0.968, 0.967, 0.979, 0.994 for class 1 to 4.

**Fig. 4:**
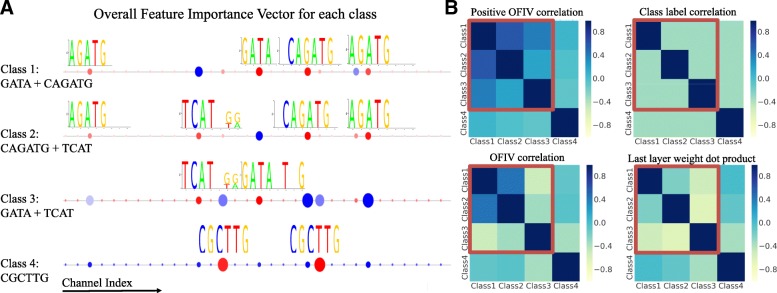
Visualization of DeepResolve in multi-task networks. **a** Overall Feature Importance Vector for Synthetic dataset II class 1 - 4. Each circle on the X-axis represents a channel, with red representing positive OFIV score and blue representing negative OFIV score. Each column corresponds to one of the 32 channels that is shared among all four classes. OFIV successfully ranks predefined sequence features as the most important features for each of the classes, while reveals ‘unfavored’ features that are used to separate a class from its competing classes. **b** Correlation matrix of class based features shows the benefit of non-negative OFIV scores. The predefined sequence pattern for each class is shown (**a**). Our proposed Class Similarity Matrix (top-left) successfully assigns high correlation to (Class1, Class2), (Class2, Class3) and (Class1, Class3) and low correlation to all pairs with Class 4. The matrix in top right corner suggest low correlation between the labels of each class. The matrix on the bottom left is the Pearson correlation of ONIV score without removing the negative terms, and the bottom right matrix is calculated by taking the cosine of the corresponding rows in last layer weight matrix. The bottom two both fail to assign higher similarity score to combinations of classes that share sequence features

Figure 4a shows the OFIV for each of the classes, and the optimal number of Gaussian mixture components of the IL score for all of the classes is one (Additional file 1: Figure S1), correctly indicating that only additive channels exist in these classes. We observe that the channels with the top OFIV (red) correctly capture the sequence determinants of the corresponding class. We observe strong negative terms (blue) in OFIVs for all classes, representing sequence patterns ‘favored’ by other alternative classes, which validates our hypothesis that the ’process of elimination’ truly exists. Figure 4b compares class similarity matrices generated by our method and using the last layer weight matrix. The non-negative OFIV correlation matrix successfully assigned higher similarity score to class 1+2, class 1+3 and class 2+3, while the other methods failed to do so. Note that for class 1+3 and class 2+3, the similarity scores estimated by the last layer weight dot product are strongly negative, suggesting that the same features will lead to the opposite predictions between these pairs of classes. While consistent with label correlation, this interpretation is contradictory to the fact that those classes are actually similar in feature composition, showing limitations of conventional methods that are based on the last layer weight. The correlation when using both positive and negative ONIV scores suggest similar pattern as the last layer weight, showing that the negative terms confounds the similarity analysis.

### Experimental datasets

We analyzed two experimental datasets to examine DeepResolve’s ability to recover biologically important features, and to discover correlation in features that might relate to mechanism.

#### Identifying key motifs in models of TF binding

We applied DeepResolve to convolutional neural networks trained on 422 Transcription Factor ChIP-Seq experiments for which the TF motifs are available in the non-redundant CORE motifs for vertebrates in JASPAR 2015 ([6, 7]) and only one motif exists for each TF. The positive set contains 101-bp sequences centered at motif instances that overlap with the ChIP-seq peaks. For each TF, the JASPAR motif for the corresponding factor (Additional file 1: Table S1) is used to identify motif instances using FIMO. The negative set are shuffled positive sequences with matching dinucleotide composition. Each sequence is embedded into 2-D matrices using one-hot encoding. We train a single-class CNN for each experiment using one convolutional layer with 16 filters of size 25 with global max-pooling, and 1 fully connected layer with 32 hidden units. The mean of the AUC for these 422 experiments is 0.937 and the standard deviation is 0.035. We then generate FIMs and OFIVs for each experiment on the last convolutional layer, and rank the filters using OFIV scores. 420 of the 422 experiments contain only additively contributing features (Additional file 1: Figure S1).We convert the top filters into position weight matrices (PWMs) and match them with known motif for the target TF using TOMTOM [28], and count how many times we hit the known motif in top 1, top 3 and top 5 filters with matching score *p*-value less than 0.5 and 0.05. We compare our method to DeepMotif ([26]), a visualization tool that generates important sequence features by conducting gradient ascent directly on the input layer. We improved DeepMotif’s initialization strategy to allow multiple random initializations instead of using an all 0.25 matrix (naming it enhanced-DeepMotif), and take the most informative 25bp fragment of generated sequences with top 5 class score. We also compared with three gradient-based methods, deepLIFT,saliency map, and its variation where the gradients are multiplied by the inputs to the neurons. However we conducted them on an intermediate layer instead of on input layer. We used all sequences from the positive training set, and took the average of scores assigned to a channel as an indication of the importance of a channel.

Shown in Table 1, our method successfully proposes known matching motifs as top 5 features in all of the 422 experiments with TOMTOM *p*-value less than 0.5, and in 421 out of 422 experiments with *p*-value less than 0.05, which outperforms enhanced DeepMotif by ∼ 3-fold. Our method also outperforms saliency map and its variation in top-1, top-3, top-5 accuracy, and outperforms deepLIFT in top-3, top-5 accuracy with TOMTOM *p*-value less than 0.5. We selected the top filter that matched a known canonical motif with lowest TOMTOM *p*-value from each experiment, and conducted Mann-Whitney Ranksum (unpaired) and Wilcoxon (paired) rank test between the ranks that DeepResolve and input-dependent methods assign to these filters. Our method is significantly better (*p*<0.000001) then the saliency map method and its variation on both tests and is comparable to DeepLIFT even though we did not refer to any input dataset when calculating our OFIVs. The distribution of optimal numbers of Gaussian mixture components for all the experiments is plotted in Additional file 1: Figure S1, where only 2 of the experiments have potentially non-additive channels. This result demonstrates that the logic for single TF binding is mostly additive and complex feature interactions such as XOR logic are unlikely. It also shows that the convolutional filters in genomic studies can capture motifs accurately by themselves, which lays a good foundation for hierarchical feature extraction and interpretation tools like DeepResolve.

**Table 1:** Top-1, top-3, top-5 accuracy in identifying matching motif for TF binding (out of 422 experiments) with similarity score (*p*-value) smaller than 0.5 and 0.05, and the paired/unpaired rank tests of the proposed ranks of best matching filters between our method and the input-dependent methods

	Top 1		Top 3		Top 5		Ranksum	Wilcoxon
TOMTOM *P*-value	0.5	0.05	0.5	0.05	0.5	0.05	*p*-values	*p*-values
DeepResolve (ours)	412	407	421	418	422	421	N/A	N/A
DeepLIFT	418	418	420	420	421	421	0.784	0.412
Saliency*activation	404	393	419	419	420	420	7.478×10^−7^	1.049×10^−9^
Saliency	388	377	417	416	420	420	4.63×10^−7^	4.63×10^−15^
enhanced DeepMotif	217	89	310	123	343	147	N/A	N/A

**Fig. 5:**
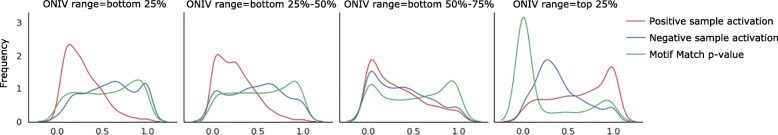
Distribution of positive sample activation level, negative sample activation level and motif matching *p*-values of filters grouped by their ONIV score ranking. We collected convolutional filters from all 422 TF binding models and group them into four groups by the ranks of ONIV score, each containing 1688 filters. Each panel represents one of the groups and the ONIV ranks increase from left to the right. The averaged activation scores across all negative and positive examples are calculated for each filter, and is normalized to [0,1] within each network. The top ranking group (right most) has high activation in positive examples while low activation in negative examples, and has the most significant motif matching pvals. This is suggesting that DeepResolve ranks highly relevant and informative filters that can separate positive and negative set well

We further analyzed the learned convolutional filters from all 422 TF binding models by visualizing their activation patterns and relevance to known motifs. We grouped them into four groups by the ranks of ONIV score and plotted the distribution of the averaged activation scores across all negative and positive examples. We also plotted the distribution of TOMTOM *p*-values of the corresponding motif for each group. As shown in Fig. 5, the top ranking group (right most) has highest activation in positive examples and lowest activation in negative examples, and has the most significant motif matching *p*-values. This suggest that ONIV successfully selected highly relevant and informative filters that can separate the positive and negative sets.

#### Identifying sequence feature sharing and class correlations in DeepSEA

We evaluated DeepResolve’s ability to discover important features and identify shared features and class similarities across distinct classes in the DeepSEA network[8], a classic multi-task convolutional network trained on whole genome data to predict 919 different features including chromatin accessibility, TF binding and histone marks across a variety of cell types. DeepSEA compresses a large training set into its parameters and thus we sought to interpret DeepSEA’s parameters to uncover biological mechanism.

**Fig. 6:**
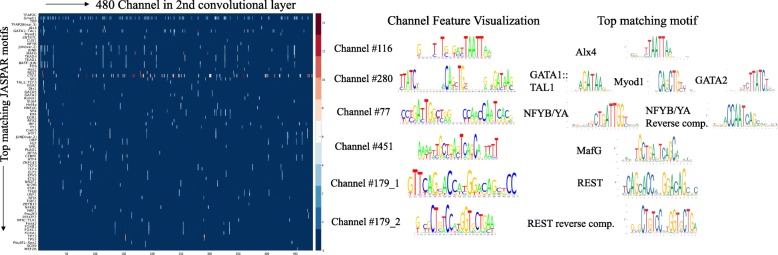
Visualization of sequence features captured by the 480 channels in 2nd convolutional layer of DeepSEA. The sequences are generated using gradient ascent (see section 1). The matrix represents -log10 of TOMTOM Q-values for each pair of channel and its top matching motifs. Each row represents a known JASPAR motif which has been ranked as top 1 matching motif for at least one of the channels. Only pairs that achieve less than 0.005 Q-value are represented with actual Q-value, and the dark blue region represents default value for low Q-values. In the right panel, the left column shows the SeqLogo visualizations of representative gradient ascent outputs of 5 of the channels, and the top matching motifs are shown in the right column. Channel 116 and 451 captures single motif of Alx4 and MafG. Channel 280 captures 3 consecutive motifs (GATA1,Myod1 and GATA2), while channel 77 captures consecutive NFYB/YA motif and its reverse compliment. Channel 179 captures either REST or its reverse compliment depending on the input sequences used for initialization

In DeepSEA, input sequences are 1000bp long, and the labels are 919 long binary vectors. The network has 3 convolutional layers with 320, 480, 960 filters, and 1 fully connected layer. We chose the input to the 3*rd* convolutional layer as *H* to generate feature importance maps, where the activation of a channel is determined by a 51bp sequence segment in the input (receptive field). We visualized the sequence features of a channel by *l*_2_-regularized gradient ascent over its receptive field to maximize the channel activation. We initialized the input with the top ten 51bp fragment from the training sequences that maximize the channel activation. We applied a heuristic thresholding to the optimized input segments and normalized them to sum up to one in each column, and used TOMTOM to compare the resulting position weight matrix with known JASPAR motifs. Figure 6 left panel shows the -log10 of the TOMTOM Q-values for each pair of channel and its top matching motifs. We discovered 218 channels that capture sequence features that match with 200 known JASPAR motifs with Q-value smaller than 0.005, and we observed channels that capture single motif, multiple motifs, consecutive motif with its reverse compliment (Fig. 6). We show that a single channel can capture both a motif and its reverse compliment depending on the input sequences, and we captures this dynamic by using multiple initializations for the gradient ascent.

**Fig. 7:**
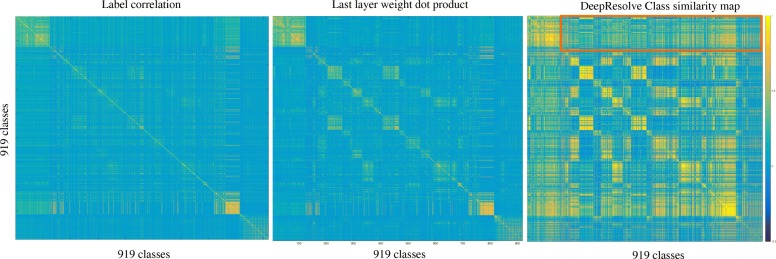
Class similarity map for DeepSEA. X and Y axis represents 919 different experiments including DNase I hypersensitivity, TF binding and histone marks across different cell types. The sub-matrix highlighted by the red box is used for DNase correlation pattern analysis in Fig. 8

We next computed a class similarity matrix based upon OFIVs and found that the resulting matrix revealed similarities between the decision functions that underlie distinct classes, even when the classes themselves were not strongly correlated. We first calculated FIVs and their weighted variances for each class. The distribution of optimal numbers of Gaussian mixture components for all the experiments is plotted in Additional file 1: Figure S1, where only 2 of the experiments have potentially non-additive channels. This indicates that the majority of the classes in DeepSEA employ additive logic where binding can be determined by the additive contribution of several motifs. We then generated a class similarity matrix as described in Section 1. Given that DeepSEA takes in 1000bp long sequences around the biological event, it captures upstream and downstream sequence context. Therefore our proposed metric measures similarities between the contextual structures of a pair of regulators, which could imply interesting correlations in functionality and mechanism. Figure 7 compares DeepResolve’s class similarity matrix with the label correlation matrix and the dot product matrix of last layer weights for all classes. DeepResolve’s class similarity matrix revealed strong correlation between pairs of TFs/histone marks/DNase hypersensitivity that do not necessarily co-appear within 200 bp or having strong last layer weight correlation, but are functionally relevant.

**Fig. 8:**
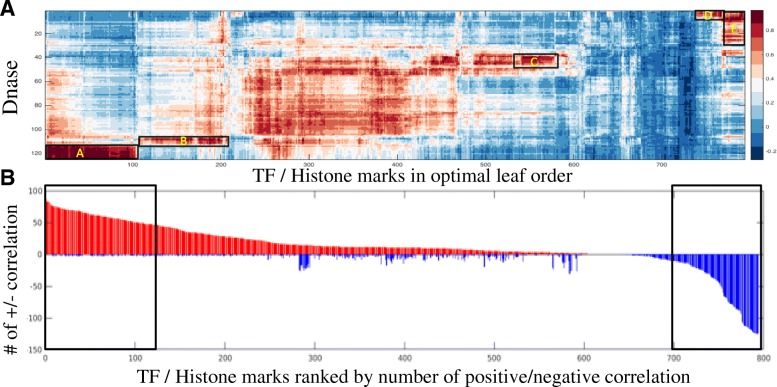
**a** Bi-clustering of TF/histone mark - DNase hypersensitivity similarity map (the highlighted box in Fig. 7), x-axis are the TF/histone mark experiments and y-axis are DNase hypersensitivity experiments across 125 different cell types. A zoom-in of the clusters can be found in Additional file 1: Figure S1. **b** Bar-plot of number of strong positive (red) and strong negative class similarity (blue) with DNase experiments for each of the TFs and histone marks. Majority of the TF/histone marks in the left box are known chromatin regulators, and majority of TF/histone marks in the right box are known transcription repressor. A zoom-in of the bar-plot can be found in Additional file 1: Figure S2

We then examined the correlation pattern between selected TF/histone marks and DNase I hypersensitivity across different cell types to explore the shared components of their decision functions. Figure 8a shows the bi-clustering result on the TF-histone mark/DNase similarity matrix. We observed clusters of TFs and histone marks sharing similar patterns, and some of them exhibit cell-type specific effect on DNase hypersensitivity (see Additional file 1: Figure S2). We collapsed the map into 1-D by calculating number of strong positive similarity (larger than 0.52, 85% quantile of all correlations) and negative similarity (smaller than 0, 15% quantile of all correlations) with DNase experiments for each TF/Chromatin mark. As shown in Fig. 8b, we characterized each TF and histone mark’s association with chromatin accessibility using these indexes. We identified groups of TFs/histone marks that are highly correlated with DNase hypersensitivity (located to the left side of the histogram), and most of them are known to be involved in Chromatin Regulation / Acetylation Pathway, e.g. CTCF, POL2, CHD1/2, PLU1(KDM5B), SMC3, RAD21, GTF2B/GTF2F1, TBP, etc., or known to be essential for transcription activation, e.g. PHF8, USF2, H3K4me2, H3K27ac. We also identified groups of TFs/histone marks that are negatively correlated with DNase hypersensitivity and observe that most of them are well-known transcriptional repressors and repressive marks, e.g. ZNF274, EZH2, SUZ12,H3K9me3, H3K27me3 (see Additional file 1: Figure S3 for detailed list of the TFs/histone marks inside the box plotted in Fig. 8).

**Fig. 9:**
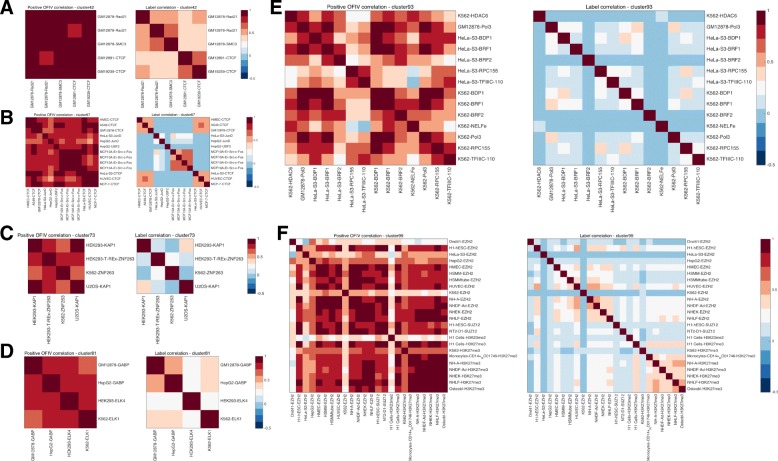
Hierarchical clustering results of 919 biological targets using correlation of positive OFIV as distance metric. Each panel represents a cluster, in which the left matrix is the sub-matrix of the class similarity map in 2nd convolutional layer(see Fig. 7) among classes in the cluster, and the right matrix is the sub-matrix of label correlation between the classes. Each of the clusters consist of TFs that are known to be interacting, such as forming a complex or cohesin (c-Fos and JunD (**b**), SMC3 and Rad21 (**a**)), co-repression (KAP1 and ZNF263 (**c**)), competing (ELK1 and GABP (**d**) or known to be essential for each other to regulate transcription (EZH2, SUZ12 and H3K27me3 (**f**)). Cluster (**e**) consists of the subunits of Pol III (RPC155) and 2 essential transcription factors for Pol III : TFIIIB (BRF1/2 and BDP1 are subunits for TFIIIB) and TFIIIC. We show that even when the label correlation is not significant, our class similarity matrix can still capture the functional relevance of the interacting TFs

Another way of utilizing the class similarity matrix is to directly use it as a metric of distance for clustering. We performed hierarchical clustering of the 919 ChIP-seq experiments and identified meaningful clusters where targets within the same cluster are known to be similar to each other, including groups of the same TF across different cell types, or groups of different TFs in same cell type (Fig. 9). We found many of the clusters consist of TFs that are known to be interacting, such as forming a complex or cohesin (c-Fos and JunD [29]; SMC3 and Rad21 [30, 31]),co-repression(KAP1 and ZNF263 [32, 33]), competing (ELK1 and GABP [34]) or known to be essential for each other to regulate transcription (EZH2, SUZ12 and H3K27me3 [35, 36];Pol III (RPC155),TFIIIB (BRF1/2 and BDP1 are subunits for TFIIIB) and TFIIIC). We contrast the result from DeepResolve with the label correlation matrix for each cluster and show that even though label correlation pick up some of the above mentioning pairs (e.g. SMC3 and Rad21), it can sometimes miss some pairs (e.g. c-Fos and JunD, KAP1 and ZNF263) while DeepResolve captures these pairs even when data from different cell types are used. We further visualize the OFIV of clusters that exhibit cell type or TF specificity, and recognize sequence features that are potentially contributing to cell type specific binding or the binding of a single TF across different cell types (see Additional file 1: Figure S4).

## Discussion

### Potential artifacts in minor cases

Our method is designed to preserve positively attributed channels when generating an ONIV. It is possible that a channel detects the existence of an input feature through reduction of activation, and a negatively attributed channels of this type can be positively contributing to the output. We visualize the information content of positive and negative weights from all convolutional filters in the 422 TF binding experiments (see Additional file 1: Figure S5), and we show that networks tend to learn more information from positively weighted evidence than negatively weighted evidence. This can be in part explained by the bias of back-propagating gradients for positively activated neurons when ReLU is used. Our observations suggest that negative-negative paths in neural networks are infrequent and thus our design choice towards biasing the positive channels is not very likely to be confounded by these paths.

We noticed that in some experiments, high ranking filters do not always match the known ground truth. While these filters may be artifacts, we found their existence highly relevant to the network and the training data and thus they should not be ignored. We analyzed the normalized activation level in the postive examples, information content and the motif matching *p*-values of all convolutional filters in the 422 TF experiments. As shown in Additional file 1: Figure S5B, there exist strongly activated filters with high information content while their *p*-value for motif matching is not significant. Moreover, we divided filters into four groups depending on the ranks that DeepResolve assigned to them, and we visualized their activation level in positive examples verses the motif matching *p*-values, colored by the information content of its positive weights. As shown in Additional file 1: Figure S5C and Fig. 5, the top ONIV ranked filters are highly activated in positive samples and have low activation in negative examples, and match known motifs with high significance. Filters located on the right top corners are strongly activated in positive training example while not matching a known motif. These could either be the result of over-fitting the training set or true patterns in the training set that are not covered by the chosen known motif. There exist some top ranking filters that are low in both activation and motif matching significance (circled in green in Additional file 1: Figure S5C), we consider this type of filters as artifacts of the visualization procedure. Among 1688 filters in the top 25% group, only 67 (less than 4%) of them belong to this type (*p*-value larger than 0.5, activation level within bottom 25%). We also found that this artifact exists in all visualization methods that we examined, 12 in DeepLIFT and 35 in saliency map.

### Intermediate layer selection for analysis

DeepResolve can learn feature contribution and interaction patterns at any layer of a network with regard to any desired output neuron, and thus it is important to select a layer for network interpretation that is informative for a specific task. We find that a good heuristic is to select a layer *L* such that its neuron activation correspond to local sequence patterns comparable to motifs. In addition, the selected layer should not be distant from an output neuron of interest. This is because additional intervening non-linear layers introduce excessive instability that can inhibit learning accurate feature interactions. For many existing networks for predicting genomic functional regulatory elements the optimal choice for analysis is the layer located between the fully connected layers and convolutional layers [6, 7]. For DeepSEA [8] which has 3 convolutional layers, we found the input to last convolutional layer is most informative. We also observed that as we pick layers that are closer to the input, the similarity matrix becomes denser because the sharing of lower level features is more likely than the sharing of higher level features. Thus picking the right layer for analyzing class similarity depends on the feature granularity desired.

### Selection of hyper-parameters

The L2 norm in the objective function for gradient ascent is essential in controlling the scale of generated feature maps. We experimented with different L2 coefficients *λ* ranging from 0.3 to 2.8 and observed that *λ* does not substantially affect the ranking of channels in general, even though the scale of generated FIVs varies with the choice of *λ*. A good heuristic for picking *λ* is to select a *λ* such that the resulting feature importance map has a norm that is comparable to the norm of mean feature map activation which can be calculated using a small set of realistic input sequences randomly sampled from the training set. We tested different step sizes including 0.1,0.01,and 0.001, and we also found that the step size of gradient ascent does not have a significant effect on the results when it is reasonably selected. It should not be so large that the objective does not increase and not so small such that the convergence rate is extremely slow. In practice we use *learning rate decay* to gradually reduce the learning rate with the number of steps.

$$lr=lr_{0}*max((step-start\_decay)^{-\alpha},min\_lr)$$

### Complex logic and feature sharing in biological problems

While we observed the DeepSEA model consists mainly of additive logic with a few non-additive channels, XOR logic may exist. The fact that XOR logic was not more obvious could be the consequence of the unbalanced training data in DeepSEA where most of the sequences have negative labels for a single class, which makes the learning of complex logic difficult. DeepResolve is defined to uncover non-additive interactions when they are present in a model, while the training of model with robust non-additive interactions can be difficult. Biological systems do contain TFs that bind differently but have partially shared features, including TFs that associate with different co-factors and shared pioneer factors[37]. In these interactions a pioneer factor opens chromatin that enables a distinct TF specific co-factor to bind. Our capability of discovering feature space correlations that are not present in label space can suggest interesting similarities between TFs that partially share a co-factor or functional role.

### Combining DeepResolve with existing tools

DeepResolve is designed to visualize how complex intermediate layer channel interactions contribute to decisions about a network task. It can be combined with any existing input-level visualization tools such as a saliency map or deepLIFT, which can provide fine-grained visualization of sequence features captured by the important channels that DeepResolve identifies. Similar work-flow was used to discover epistatic feature interactions [38]. The use of DeepResolve can ease the computational burden for input-space visualization tools by reducing the number of layers and the length of receptive field for traditional methods which can lead to better location specific and more accurate visualizations.

## Conclusions

DeepResolve is a gradient ascent based method that summarizes feature importance maps for visualizing and interpreting a network’s behavior in feature space that is reference input free. DeepResolve visualizes the complex combinatorial interactions of lower level features that are crucial to model decision making. It also recovers feature space similarities between poorly correlated classes which may suggest shared biological mechanism. It is compatible to existing methods in discovering important sequence features and provides complimentary insights.

## Additional file

Additional file 1 Supplementary Figures S1-S5, Supplementary Table S1. (PDF 10,216 kb)

## Data Availability

The DeepSEA datasets can be downloaded from http://deepsea.princeton.edu/help/. The TF binding datasets can be downloaded from http://gerv.csail.mit.edu/deepresolve/data. The JASPAR motifs used in the analysis can be found in: http://gerv.csail.mit.edu/deepresolve/JASPAR_CORE_vertebrates_nonredundant_20151026. The other datasets used and/or analysed during the current study and the code for DeepResolve are available in https://github.com/lgsaber/DeepResolve.;

## Abbreviations

BIC: Bayesian information criterion
CNN: Convolutional neural network
FIM: Feature importance map
FIV: Feature importance vector
IL: Inconsistent level
OFIV: Overall feature importance vector
PWM: Position weight matrix
TF: Transcription factor
