# Supplementary material for: Visualizing complex feature interactions and feature sharing in genomic deep neural networks
Source: BMC Bioinformatics. 2019 Jul 19;20:401. doi: 10.1186/s12859-019-2957-4 (PMC6642501; doi:10.1186/s12859-019-2957-4)

Supplementary Figure S1 Distribution of optimal number of Gaussian mixture component of variances in each set of experiments. 1 means all the channels are linearly contributing to the class, whereas a value larger than 1 means some of the channels are potentially non-linear. Consistent with the ground truth, for Synthetic I all the classes have both linear and non-linear channels, and for Synthetic II all the classes only have linear channels. For the networks trained on 422 TF binding datasets, 420 only have linear channels. For DeepSEA, 917 out of the 919 classes only have linear channels.

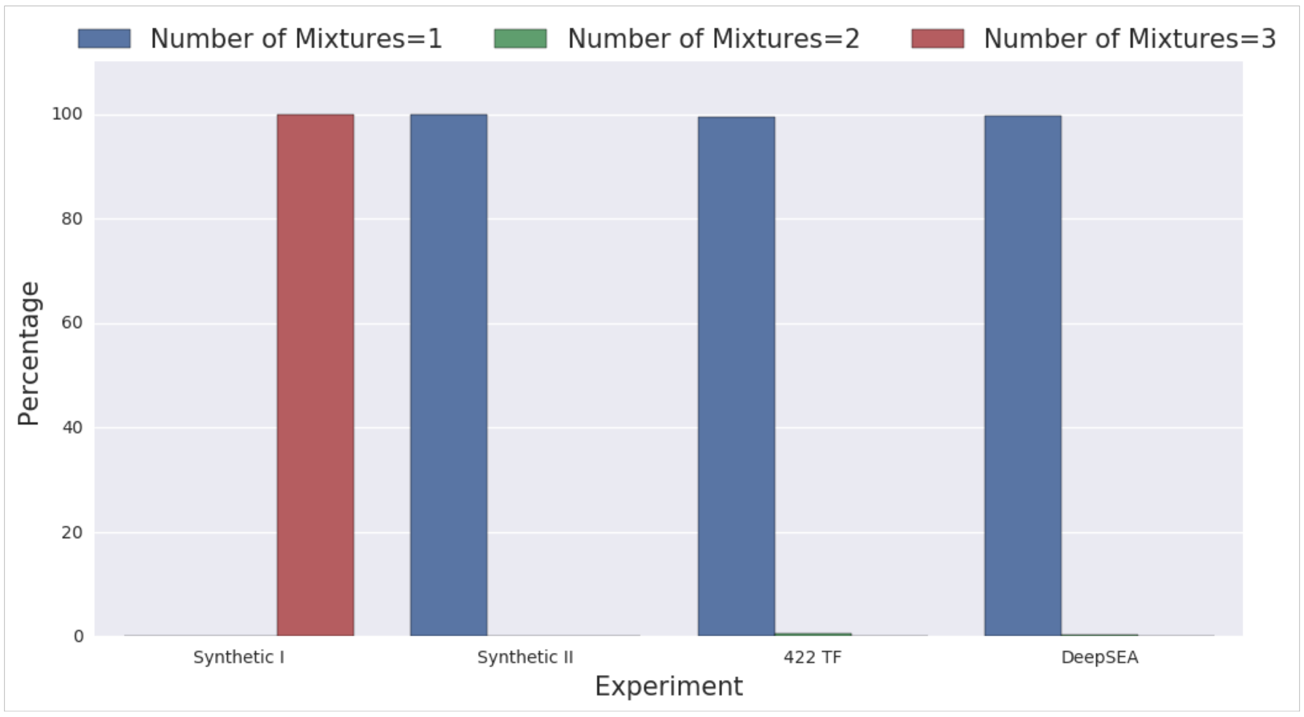

Supplementary Figure S2 Clustering results of positive OFIV correlation between TF/Histone marks and DNase hypersensitivity in different cell types. Clusters are generated using hierarchical agglomerative clustering on y and x dimensions. Cluster A, B, C, D indicate cell type specific effect of corresponding TF/Histone marks in GM cells, K562 cells, HepG2 cells and h1-hESC cells, while cluster E indicate binding patterns of NRSF and Znf143 across a set of different cell types is similar to that of DNase hypersensitivity across a wide set of different stem cells.

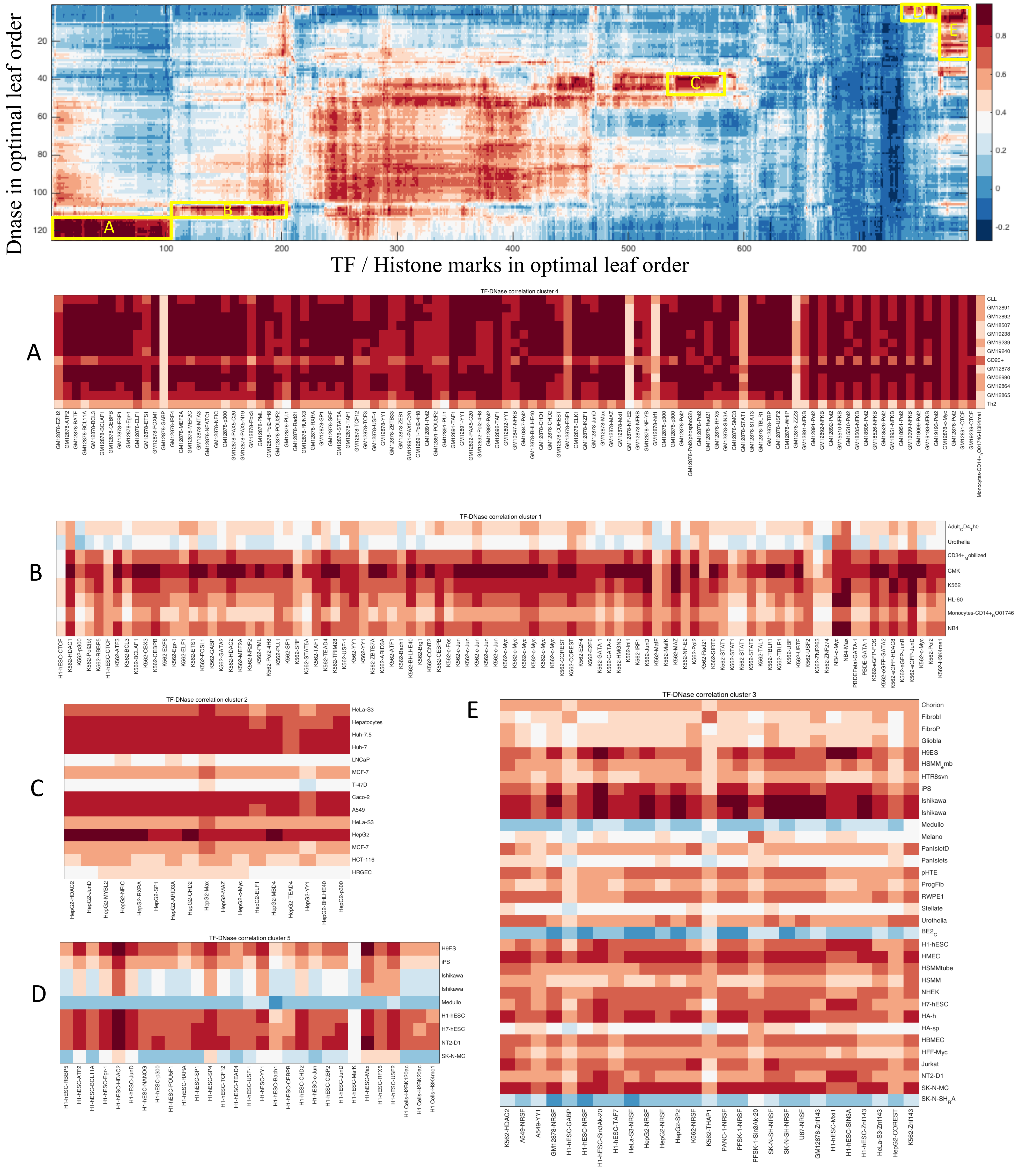

Supplementary Figure S3 Zoom-in of the top 120 entries (A) and bottom 100 entries (B) in the bar-plot in figure 6B. The list of TF/Histone marks is shown in the x-tick label. The number of strong positive correlation (>=0.52) to DNase hypersensitivity experiments is plotted in red bars, and the number of negative correlation is plotted in blue bars. The majority of the TF/Histone marks in box A are known to be involved in Chromatin Regulation / Acetylation Pathway or essential for transcription activation, while the majority of the TF/Histone marks in box B are known transcriptional repressors.

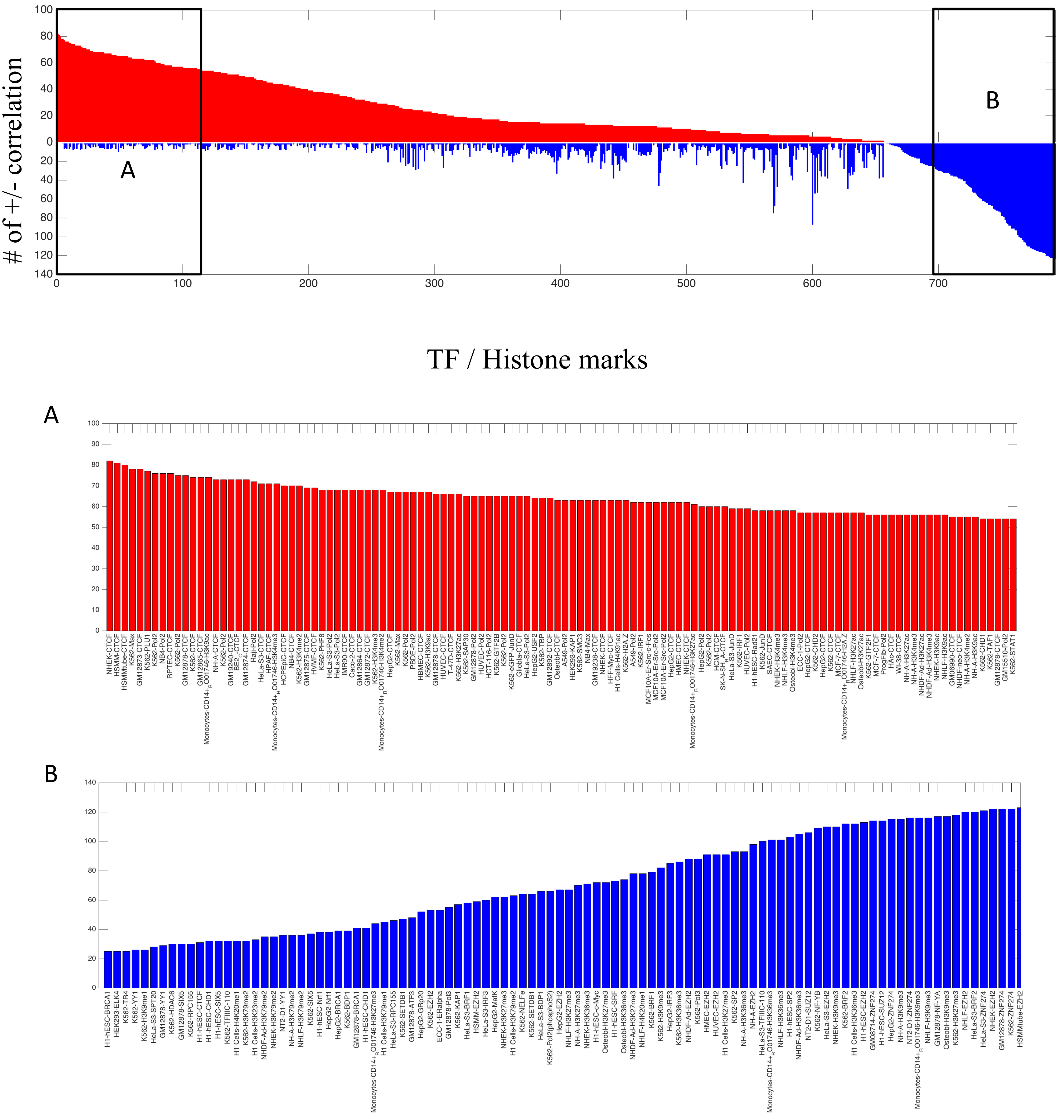

Supplementary Figure S4 Visualization of OFIVs of classes that are clustered together using hierarchical clustering results of all 919 biological targets using correlation of positive OFIV as distance metric. Each column represents a channel in the 2nd convolutional layer, which captures 51bp sequence features. A brighter color indicate higher OFIV value. A and B panel presents cell type specific binding in GM12878 and HepG2, while C and D panel presents TF specific binding across different cell types for MafK and Znf274. Sequence features generated by gradient ascent of the potentially important channels are plotted below the channel (only the most informative fragment of the 51bp feature is presented).

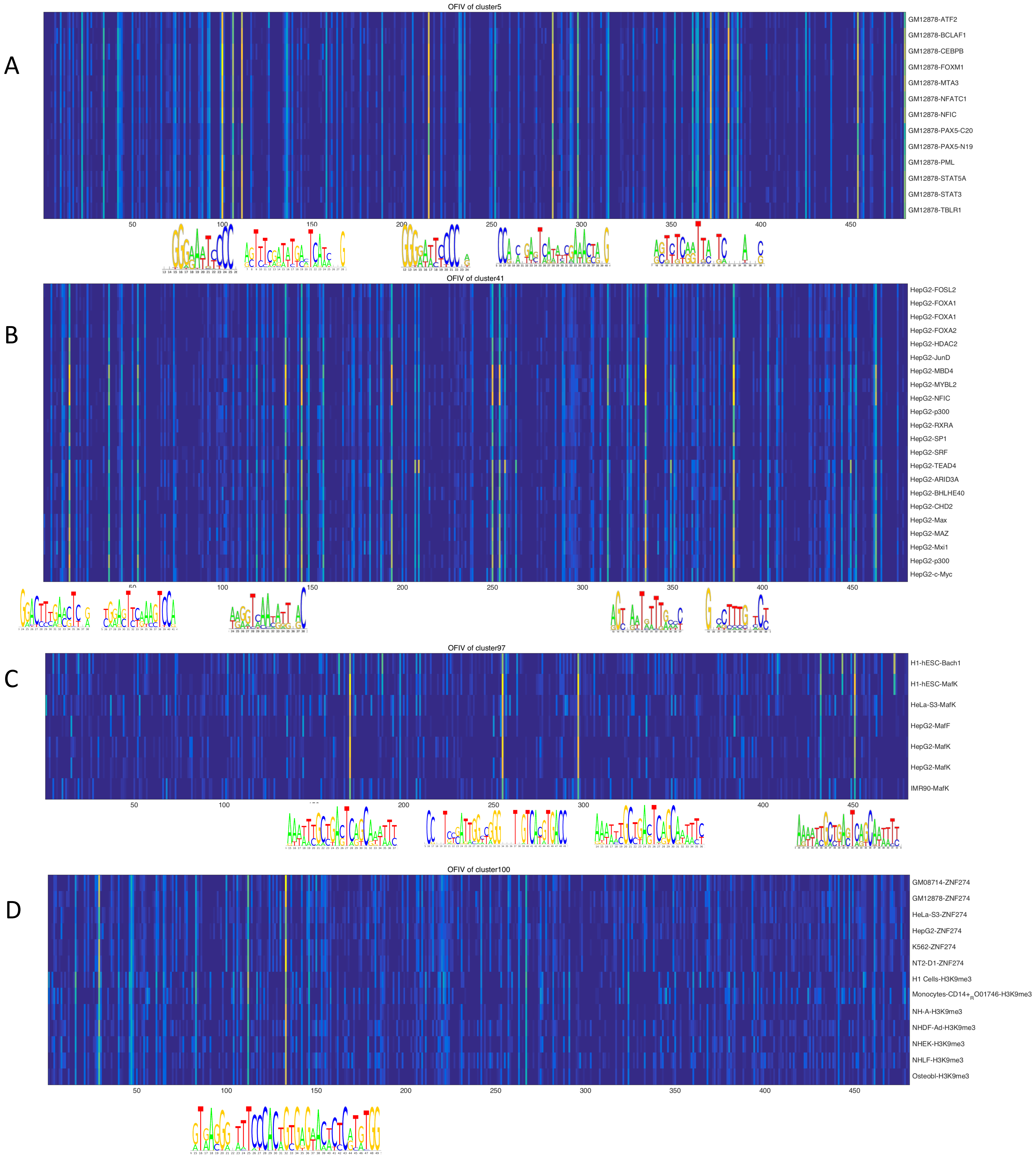

Supplementary Figure S5 (A) Distribution of information content of positive and negative weights in all filters in the 422 TF binding models. The convolutional filters tend to learn more information through positive weights. (B) Positive weight information content verses the TOMTOM p-value with known motif for all convolutional filters colored by the activation level in positive examples. There exist filters that do not match known motif while being strongly activated in positive samples and containing high information contents. (C) Positive sample activation verses motif matching p-values for filters grouped by their attribution scores. Filters with low activation and high p-values are considered artifacts in the visualization procedures. We found that artifacts exists in both our method and the baseline methods but only with a small portion.

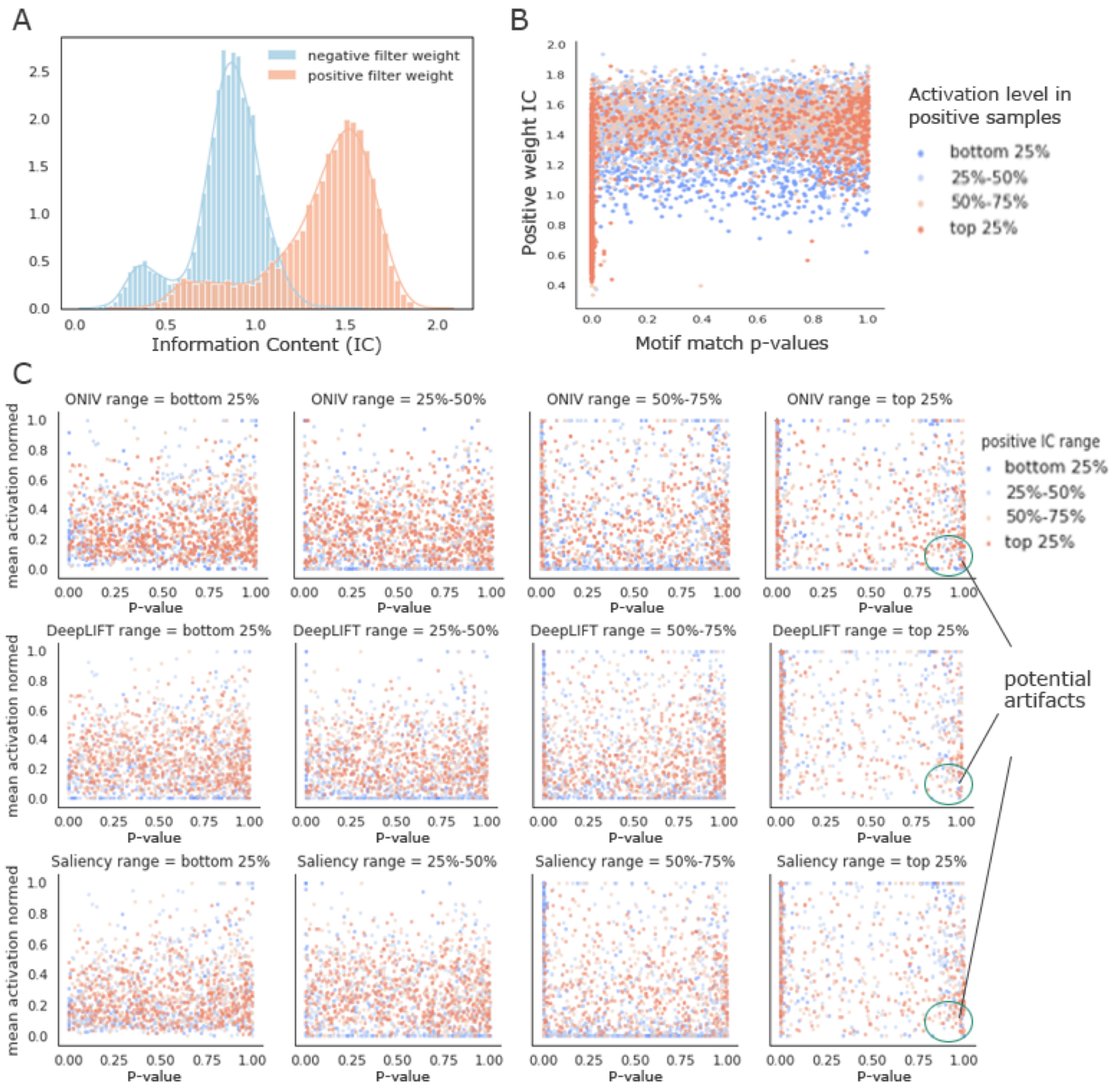

Supplement: Supplementary file 1 — Supplementary Figures S1-S5, Supplementary Table S1. (PDF 10,216 kb) [file 12859_2019_2957_MOESM1_ESM.pdf]
